# Discovery of Novel Noncovalent KRAS G12D Inhibitors through Structure-Based Virtual Screening and Molecular Dynamics Simulations

**DOI:** 10.3390/molecules29061229

**Published:** 2024-03-10

**Authors:** Zhenya Du, Gao Tu, Yaguo Gong, Xiangzheng Fu, Qibiao Wu, Guankui Long

**Affiliations:** 1 State Key Laboratory of Quality Research in Chinese Medicine, Dr. Neher’s Biophysics Laboratory for Innovative Drug Discovery, Macau Institute for Applied Research in Medicine and Health, Faculty of Chinese Medicine, Macau University of Science and Technology, Macao 999078, China2009853qct30001@student.must.edu.mo (G.T.); gongyglab@gmail.com (Y.G.); fxz326@hnu.edu.cn (X.F.); 2 Teaching and Research Department of Public Medical Courses, School of Nursing, Guangzhou Xinhua University, Guangzhou 510520, China; 3 School of Materials Science and Engineering, National Institute for Advanced Materials, Renewable Energy Conversion and Storage Center (RECAST), Nankai University, Tianjin 300350, China

**Keywords:** KRAS^G12D^, noncovalent inhibitor, virtual screening, molecular dynamics

## Abstract

The development of effective inhibitors targeting the Kirsten rat sarcoma viral proto-oncogene (KRAS^G12D^) mutation, a prevalent oncogenic driver in cancer, represents a significant unmet need in precision medicine. In this study, an integrated computational approach combining structure-based virtual screening and molecular dynamics simulation was employed to identify novel noncovalent inhibitors targeting the KRAS^G12D^ variant. Through virtual screening of over 1.7 million diverse compounds, potential lead compounds with high binding affinity and specificity were identified using molecular docking and scoring techniques. Subsequently, 200 ns molecular dynamics simulations provided critical insights into the dynamic behavior, stability, and conformational changes of the inhibitor-KRAS^G12D^ complexes, facilitating the selection of lead compounds with robust binding profiles. Additionally, in silico absorption, distribution, metabolism, excretion (ADME) profiling, and toxicity predictions were applied to prioritize the lead compounds for further experimental validation. The discovered noncovalent KRAS^G12D^ inhibitors exhibit promises as potential candidates for targeted therapy against KRAS^G12D^-driven cancers. This comprehensive computational framework not only expedites the discovery of novel KRAS^G12D^ inhibitors but also provides valuable insights for the development of precision treatments tailored to this oncogenic mutation.

## 1. Introduction

The Kirsten rat sarcoma viral proto-oncogene (KRAS) is a gene that is frequently mutated in human cancers. It belongs to the RAS family of genes, which also includes H-RAS and N-RAS. KRAS mutations are found in approximately 30% of human cancers, making it one of the most common oncogenes [1]. It is often single-point mutations that result in a change in the amino acid sequence of the encoded protein. The most common KRAS mutation is the substitution of a single amino acid at position 12, which alters GTPase activity. For example, KRAS^G12D^ is a specific mutation of the KRAS gene that involves a substitution of the amino acid glycine (G) at position 12 with aspartic acid (D). KRAS mutation results in a constitutively active form of the KRAS protein, which leads to uncontrolled cell growth and division. This mutation is often associated with aggressive and treatment-resistant cancers, making it an important target for cancer research and drug development. It has been found in a variety of cancers, including pancreatic cancer, colorectal cancer, and non-small cell lung cancer (NSCLC).

Because KRAS is a small GTPase protein that is highly conserved and has a complex structure, it is difficult to target it with small molecule inhibitors. In addition, KRAS is part of a signaling network that is tightly regulated and has many redundant components, making it challenging to develop inhibitors that can specifically target KRAS without disrupting other cellular processes. However, recent research has identified potential therapeutic strategies that target KRAS or its associated proteins, providing hope for the development of effective KRAS-targeted therapies. For example, the successful development of covalent inhibitors indicated that the mutated cysteine residues in KRAS^G12C^ leveraged a binding pocket in the switch II domain, leading to the creation of clinically effective small molecule inhibitors for patients with this mutation [2,3,4]. These findings suggest that the switch II binding pocket present in all KRAS proteins might be a plausible surface for the development of additional KRAS inhibitors. 

KRAS^G12D^ is the most common KRAS mutation with an incidence rate of approximately 2.5 times higher than the G12C mutation, but there are currently no approved targeted drugs. It has been considered a more challenging target than KRAS^G12C^ as it lacks a reactive residue proximal to the switch II binding pocket; thus, novel approaches for developing selective inhibitors with substantial affinity and drug-like potency are needed [5]. Additionally, the G12D mutation introduces a negatively charged aspartic acid residue into the KRAS protein, which can destabilize the active state of the protein and increase the difficulty of designing inhibitors that can bind effectively [6]. Furthermore, the extent of oncogene addiction to KRAS^G12D^ in cancers housing this mutation remains incompletely understood, posing a critical question for the effective development of inhibitors and therapeutic regimens directed at this particular mutation. 

**Figure 1:**
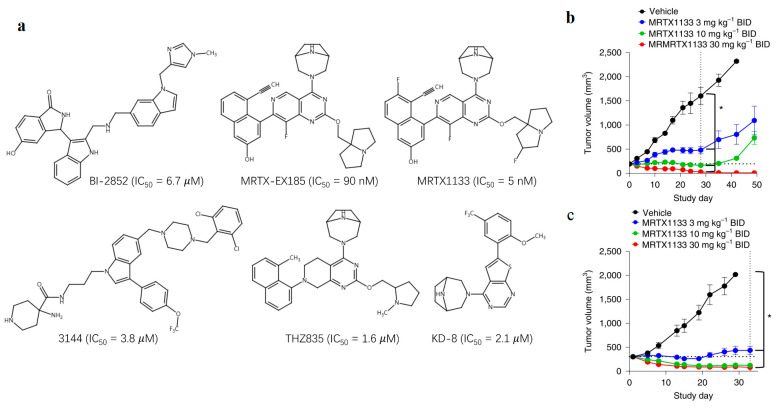
(**a**) The chemical structures of known inhibitors of KRAS^G12D^. (**b**) Normalized active RAS in tumor lysates from mice bearing Panc 04.03 xenografts treated with vehicle or MRTX1133 administered IP at the doses indicated plus one dose the next day. Brackets with * indicates statistical significance versus vehicle control using one-way ANOVA (* *p* < 0.05). (**c**) Tumor growth inhibition after IP administration of MRTX1133 BID to mice bearing MRTX1133 Panc 04.03 xenografts at the doses indicated; Brackets with * indicates tumor volumes were statistically significant versus vehicle using a two-tailed Student’s *t*-test (* *p* < 0.05). (**b**,**c**) were adapted from Ref. [9].

Due to the absence of proximal reactive residues, employing non-covalent interactions such as hydrogen bonds (HBs) or salt bridges [7] emerges as a viable strategy for designing inhibitors targeting KRAS^G12D^. In alignment with this approach, several compounds, including MRTX1133 [8,9], BI-2852 [10,11], MRTX-EX185 [12], compound **3144** [13], and TH-Z835 [7] have been developed, as shown in Figure 1. Among them, MRTX1133 has been confirmed in vivo [14]. HRS-4642 developed by Hengrui Pharmaceuticals shows an initial positive result in the clinic (NCT05533463) [15]. ASP3082, developed by Astellas Pharma, exhibits remarkable anti-tumor activity in KRAS^G12D^ mutated cancer models (NCT05382559) [16,17]. However, the details of targeting information for HRS-4642 and ASP3082 are missing. Other inhibitors, such as RMC-9805, LUNA18, QTX3034, and UA022, are still in the very early stage [18]. The success of these compounds underscores the potential efficacy of non-covalent inhibitors as a solution for targeting the KRAS^G12D^ mutation.

Computational methodologies have played a significant role in understanding and targeting KRAS^G12D^. Vatansever et al. have employed long-term Molecular Dynamics (MD) simulations and elucidated the altered structure and dynamics of the G12D mutant protein, providing insights into its destabilized active state and challenges for inhibitor binding [6]. Structure-based virtual screening, in conjunction with molecular dynamics simulations, offers an effective strategy for developing potential compounds capable of disrupting protein–protein interactions and modulating the abnormal activity of oncogenic KRAS mutants. Using a combinatorial virtual screening method, Li et al. discovered the novel thieno[2,3-d]pyrimidine analogs as KRAS^G12D^ inhibitors, which displayed potent antiproliferative activity on KRAS^G12D^ mutated cancer cell lines, especially compound KD-8 [19]. Through biological evaluation and structure-based virtual screening, Wang et al. identified four potent and noncovalent KRAS^G12D^ inhibitors [20]. Structure-based drug design (SBDD) has demonstrated its viability as a means of reducing expenses and time while performing research and optimization in drug discovery and development.

This study aimed to employ a comprehensive computational approach that combines structure-based virtual screening and molecular dynamics simulations to discover and characterize novel noncovalent inhibitors targeting the KRAS^G12D^ mutation. More than 1.7 million molecules from the ChemDiv (version 2022) and Specs (version 2022) were virtually screened, and the top 20 molecules were selected for further ADME verification. Finally, the top 17 molecules were subjected to molecular dynamics simulations to investigate the targeting strength and stability of the KRAS^G12D^ mutated protein. By interrogating the dynamic behavior, binding kinetics, and specificity of the identified inhibitors, this investigation provides crucial molecular insights essential for the development of precision therapies tailored to KRAS^G12D^-driven malignancies.

## 2. Results and Discussion

### 2.1. Virtual Screening of Compounds against KRAS^G12D^

**Figure 2:**
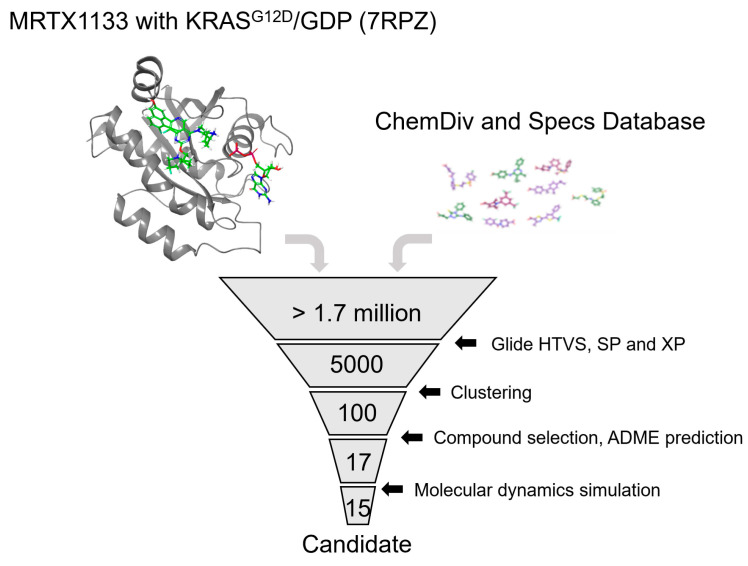
The workflow of virtual screening adopted in this study.

**Figure 3:**
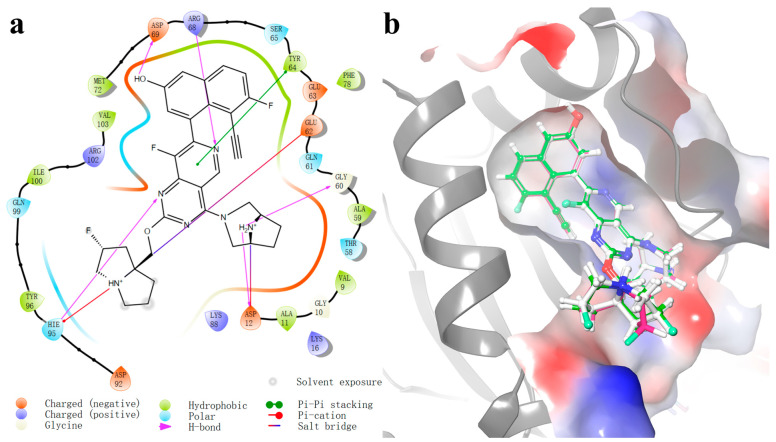
(**a**)The 2D ligand and protein interactions of the KRAS^G12D^-MRTX1133 complex docked by Glide XP; (**b**) binding modes of the KRAS^G12D^-MRTX1133 complex. The docking conformations of Glide SP (green, GScore = −13.53) and XP (pink, GScore = −12.24), and the actual conformation (white, PDB ID: 7RPZ) of the co-crystallized ligand MRTX1133.

The high-resolution X-ray structure of KRAS^G12D^ (PDB ID: 7RPZ) in complex with the ligand MRTX1133 was downloaded from the protein data bank (PDB) database [8]. More than 1.7 million compounds in the chemical database ChemDiv and Specs were screened followed by the workflow shown in Figure 2. Prior to molecular docking, the validation of the docking protocol was imperative. In this study, we used the crystal structure of the KRAS^G12D^-MRTX1133 complex (PDB ID: 7RPZ) as the template for validation. The co-crystallized ligand MRTX1133, which is used as a reference in this study, was re-docked into the active site of KRAS^G12D^ (Figure 3), revealing an exemplary correspondence between the docking conformation of MRTX1133 and its actual conformation at the active site. The root mean squared displacement (RMSD) was calculated to be 1.19 Å. The main contribution of the displacement originates from the rotation of 2-fluoropyrrolizidine. This alignment attests to the robust reliability of the employed docking methodology, thus establishing its suitability for subsequent virtual screening endeavors.

**Figure 4:**
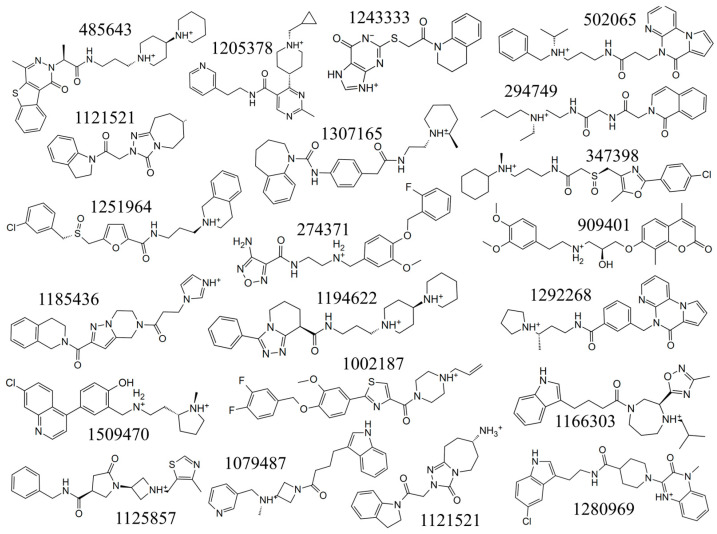
Chemical structures with charges of 20 hit compounds from Glide XP docking protocol.

Based on the docking scores and visual inspection, 20 compounds were selected as potential candidates. The chemical structures of these compounds are shown in Figure 4. The corresponding docking scores of these 20 compounds are listed in Table 1. All the 20 listed compounds have docking scores varying between −8.00 kcal/mol and −10.11 kcal/mol, which indicates that all these compounds could bind the protein efficiently. Among these compounds, compound **485643** displayed the lowest docking score of −10.11 kcal/mol, but still fell a little short of the reference MRTX1133, which has a docking score of −12.18 kcal/mol. Compound **909401** includes an aryl-oxy-propanolamine pharmacophore, which is a typical β-blocker. This indicates that compound **909401** may have the side effects of lowering blood pressure and reducing heart rate.

### 2.2. Compounds with ADME Properties against KRAS^G12D^

We calculated the ADME properties of the 20 hit compounds obtained from the virtual screening workflow using QikProp (version 5.5). The ADME analysis yielded significant properties, such as the octanol/water partition coefficient, aqueous solubility, Caco-2 cell permeability, IC_50_ value for the blockage of HERG K^+^ channels, MDCK cell permeability, and human oral absorption. The evaluated scores are listed in Table 2. According to the value of MRTX1133, the criteria for the use of inhibitors for the octanol/water partition coefficient log P, aqueous solubility log S, Caco-2 cell permeability, apparent MDCK cell permeability, and human oral absorption were set to −2.0 to 6.5, −6.5 to 0.5 mol/L, >50 nm/s, >40 nm/s and >60%, respectively. The thresholds were adopted from the QikProp manual. As the calculated logS of MRTX1133 is −6.77, which is outside of the recommended region, then we change the allowed range to −6.8–0.5. Compounds **1251964**, **347398**, and **274371** were subsequently excluded. Therefore, based on the molecular interaction and ADME properties analysis, the 17 hit compounds were finally chosen for MD simulation.

### 2.3. MD Simulation of Ligand-Protein Complex

**Figure 5:**
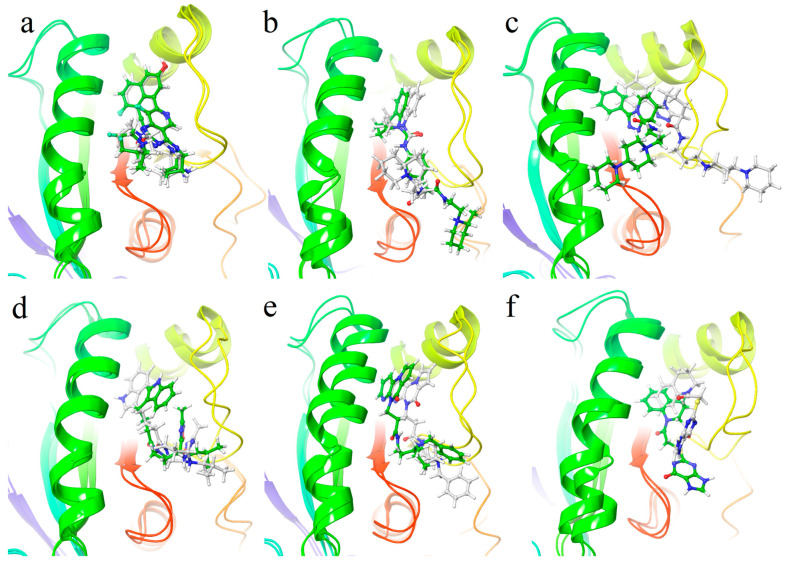
The initial (white CPK representation) and the final (at 200 ns, shown in green CPK representation) poses at the docking pocket of MRTX1133 (**a**), **1307165** (**b**), **1194622** (**c**), **1166303** (**d**), **502065** (**e**), and **1243333** (**f**). The protein is shown as a ribbon colored by residue position.

Next, 17 selected compounds identified via structure-based virtual screening were docked into the KRAS^G12D^ binding site and their MD simulations were performed. MRTX1133 was used as the positive control. The initial and the final pose of the molecular dynamics were compared to testify whether the compounds are firmly bound as shown in Figure 5, Figures S1 and S2 (available in the Supplementary Materials). For the MRTX1133, the change of pose before and after the MD simulation is very small: the backbone in the deep cavity is kept, and only the 2-fluoropyrrolizidine group is slightly variated. The groups in the cavity of compounds **1307165**, **502065**, and **294749** were slightly shifted. While the other groups in the half-open space lack anchor, they behave like a tail wagging. The geometries of other compounds demonstrated significant change although they are still in the docking pocket. It must be mentioned that compounds **1125857** and **1205378** escape from the docking pocket, which means these two compounds may not be good KRAS^G12D^ inhibitors.

To quantitatively evaluate the configuration change during MD simulations, the RMSD of KRAS^G12D^, ligands, and their complex were carried out. The RMSD of the complex of KRAS^G12D^ and MRTX1133 is approximately 3.30 Å as shown in Figure S3a. However, the corresponding RMSD of **1307165** is approximately 2.10 Å, which is even smaller than MRTX1133, which is inconsistent with the initial and the final pose comparisons of MRTX1133 and **1307165** as aforementioned. We argue that the RMSD of the complex is unable to demonstrate whether the compound is firmly bound to KRAS^G12D^ because the RMSD mainly originates from KRAS^G12D^ as KRAS^G12D^ is much larger than the ligand. As shown by the RMSD (black line) of KRAS^G12D^ and the RMSD (green line) of the complex in Figures S3–S5, the RMSDs of the complex and KRAS^G12D^ are almost the same for every compound. Then, the RMSD of the ligand (after alignment) was carried out. It is 0.34 Å for MRTX1133, which is smaller than that for all hit compounds. Among these compounds, **909401** and **485643** give relatively smaller ligand RMSDs of 0.97 and 0.97 Å, respectively. The RMSD of compound **1307165** displays the largest RMSD of 3.56 Å due to the wagging tail group. It is found that even the compounds **1125857** and **1205378** have left the docking pocket and their RMSD are 2.46 and 2.56 Å due to alignment. Considering that the docking pose is the relative position of the ligand and the docking pocket (residues 3–13 and 58–101), the RMSD of the ligand and the docking pocket may be a good representation. Again, because the docking pocket is much larger than the ligand, the deviation may be mainly attributed to the docking pocket. Therefore, the ligand RMSDs were calculated by aligning the configuration to the docking pocket, shown as the blue line, and labeled as MOL-PKT in Figures S3–S5. The RMSD of MRTX1133 is approximately 0.70 Å. Compounds **502065**, **909401**, and **294749** demonstrated RMSDs less than 3.00 Å and could be considered as firmly bound. Compounds **1307165**, **1166303**, **485643**, **1292268**, and **1509470** also show small RMSD within 6.00 Å. Their docking should be stable. Further analysis finds that the wagging tail of compound **1307165** is due to the unpreferable configuration of the initial docking pose. In the initial pose, both the amines in the tail group demonstrate no interaction with the pocket. After 10 ns relaxation during MD simulation, the tail flips to the space near Asp12, and then two HBs and one salt bridge form. There is no obvious structure change after 10 ns. Compounds **1194622**, **1243333**, **1292268**, **1185436**, **1002187**, **1121521**, **1280969**, and **1079487** exhibit increasing RMSD or relatively large RMSD. This finding indicates the binding of these compounds to proteins may not be stable. Longer MD simulations are required for further verification. Surprisingly, compounds **1125857** and **1205378** produced very large and increasing RMSDs, which suggests that the ligand is leaving, and then left the docking pocket. Therefore, these two compounds are not good KRAS^G12D^ inhibitors.

The average binding free energy (ΔG) of these 17 compounds and MRTX1133 were evaluated and are shown in Table 3. MRTX1133 has the most negative average binding free energy (ΔG) of −60.58 kcal/mol, followed by compound **1307165** with a ΔG of −41.94 kcal/mol, which is 70% of that of MRTX1133. Compounds **1194622** and **1166303** also show relatively large ΔG values of −31.71 kcal/mol and −30.47 kcal/mol. All other compounds demonstrate medium ΔG except **1125857** and **1205378**. These two compounds not only exhibit very lower |ΔG| < 10 kcal/mol, but also have increasing RMSD during MD, indicating unstable binding. Generally, all other compounds demonstrated larger |ΔG| > 10.00 kcal/mol, and small structure and free energy fluctuations during MD. Counting the binding energy and stability observed in MD simulations, 15 molecules, especially **1307165**, have the potential to be effective inhibitors.

**Figure 6:**
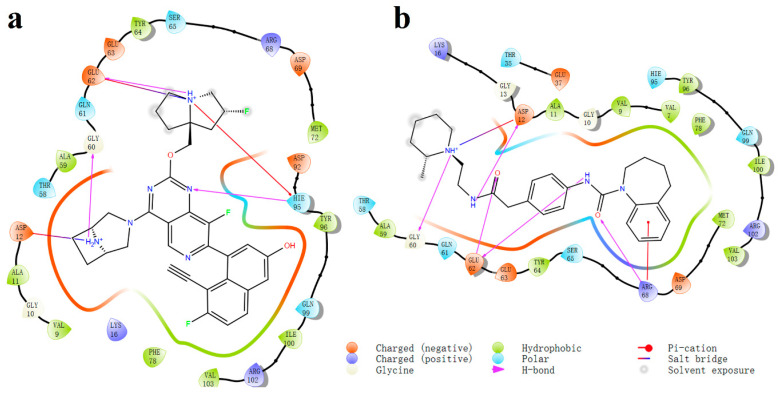
(**a**) The interaction map between MRTX1133 and the KRAS^G12D^ at 180.0 ns; (**b**) The interaction map between compound **1307165** and KRAS^G12D^ at 159.6 ns. The frame used to plot the interaction is the frame with the greatest number of HBs in the dominant group.

To determine the reason for the lower binding energy compared to MRTX1133, interaction maps were drawn. The saved MD trajectories were grouped into three groups using the epsilon clustering method. The group with the greatest number of frames was chosen as the dominant group, which should be the most representative. The frame with the greatest number of HBs in this group was used to construct the interaction map. Figure 6a illustrates the ligand interactions with the protein, 6 HBs were observed for MRTX1133. These HBs tightly bind the ligand to Asp12-N(H_2_)R_2_^+^, Gly60-N(H_2_)R_2_^+^, Glu62-N(H)R_3_^+^ (agree with X-ray crystal structures with KRAS^G12D^/GDP) [8], Asp69, and Hie95-N(H)R_3_^+^. The HB between Asp69 and the OH group of MRTX1133 exists in all the MD configurations. The HBs of Hie95 and Gly60 are in 95% and 94% MD configurations. In addition, the Hie95 could also form HB with the F in MRTX1133 in 43% configurations. The NH_2_^+^ site above the seven-element ring forms two HBs with Asp12, each show in 84% and 79% configurations. Two HBs on Glu62 with N between five-element ring, exhibit in 66% and 59%. The strong binding of MRTX1133 with KRAS originated from these abundant HBs rather than covalent bonds. Five HBs are observed for compound **1307165** binding with Asp12 and Glu62. A pi-cation interaction with Lys16 is also observed (Figure 6b). The strongest bond is the two HB between Arg68 and the O between two N atoms, with an occurrence of 79% and 75%, followed by the HB between Gly60 and N with an occurrence of 68%. HBs between Asp12 with the two HN of ligand have been observed in 31% and 30% of MD trajectory. There are other HBs but their occurrence is lower than 20%. The slightly fewer HB and lower occurrences in compound **1307165** indicate its binding energy will be slightly lower than MRTX1133.

Besides HBs, stronger binding salt bridges are also present. It is found that there are two salt bridges (Asp12-N(H_2_)R_2_^+^ and Glu62-N(H)R_3_^+^) in the MRTX1133. Only one salt bridge (Asp12-N(H)R_3_^+^) is observed for compound **1307165**. In the interaction map, for compound **1194622**, only two HBs (Glu62-N(H)R_2_ and Hie95-OH) are present between the ligand and the KRAS^G12D^, no salt bridge is observed. In the full MD process, HBs of Hie95-OH, Glu62-N(H)R_2_, Asp92-N(H)R_3_^+^, Asp92-N(H)R_3_^+^ (different O in Asp92), Glu62-N(H)R_3_^+^, and Glu62-N(H)R_3_^+^(different O in Glu62) are observed with occurrences of 62%, 53%, 50%, 42%, 30%, and 26%, respectively. Generally, compound **1307165** has one salt bridge and one hydrogen bond less than MRTX1133, leading to 20 kcal/mol higher in binding energy. Compound **1194622** has two salt bridges less than MRTX1133, leading to a 30 kcal/mol higher binding energy. We could conclude that a salt bridge contributes 15 kcal/mol and a hydrogen bond contributes 5 kcal/mol to the binding energy. Based on the structure-dependent interaction analysis, we found that most interactions between ligand and KRAS^G12D^ are dominated by KRAS^G12D^ residue-N(H_X_)R_4-x_^+^ of ligand (for salt bridge and hydrogen bond) or KRAS^G12D^ residue-N(H_X_)R_3-x_ of ligand (for hydrogen bond). Therefore, we conclude that a valid KRAS^G12D^ should have abundant amine groups and be easily charged.

## 3. Method

Figure 2 illustrates the virtual screening workflow, which has demonstrated efficacy in the identification of inhibitors in diverse enzyme systems, such as HPK1 and LRRK2. Notably, each stage of the virtual screening and molecular docking process was meticulously executed using the Schrodinger software (version 2018-1). The ChemDiv and Specs chemical databases comprising more than 1.7 million compounds were subjected to screening based on the KRAS^G12D^ receptor. The compounds were first filtered according to Lipinski’s parameters to ensure the drug-likeness of the potential inhibitor compounds. Based on the docking scores and visual inspection, the top 17 compounds were selected for further MD investigations.

### 3.1. Preparation of Receptor and Ligands

The structure of KRAS^G12D^ in complex with MRTX1133 (PDB ID: 7RPZ; resolution: 1.30 Å) was obtained from the Protein Data Bank (https://www.rcsb.org/ (accessed on 1 January 2022)) [8]. The protein preparation wizard in the Schrödinger suite (version 2021) was utilized to introduce hydrogen atoms, eliminate all water molecules, assign charges, and determine protonation states at pH 7.4. Subsequently, the structures were optimized using the OPLS-2005 force field. The prepared structure was employed to generate a grid using the Glide module of Schrödinger, with MRTX1133 chosen as the center, and the grid box dimensions set at 20 Å × 20 Å × 20 Å. For virtual screening, more than 1.7 million molecules from the ChemDiv (version 2022) and Specs (version 2022) databases constituted the screening library. All the compounds were imported into the Ligprep module of Schrödinger for ligand preparation. This involved generating ionization states of molecules at pH 7.0 ± 2.0 using Epik (version 4.3), creating stereoisomers, and generating one low-energy conformation per ligand. The ligand structures were downloaded from the official website in the format of structure data file (SDF), in which important chirality has been specified. The specified chirality is retained. Other chirality centers without specification were created.

### 3.2. Structure-Based Virtual Screening

A multistage virtual screening process was employed to identify potential lead molecules targeting KRAS^G12D^. Following Lipinski’s rule of five filtering steps and removal of reactive functionalities, three sequential docking steps were implemented using the Schrödinger Glide module: high-throughput virtual screening (HTVS), standard precision (SP), and extra precision (XP). A lower docking score indicated better binding affinity between KRAS^G12D^ and ligand. At each stage, the top 10% of docked molecules were selected for further refinement. This yielded a final pool of 5000 diverse candidates, identified through k-means clustering based on hashed fingerprints. Visual inspection of KRAS^G12D^-ligand binding modes then narrowed down the selection to 20 compounds for further analysis.

### 3.3. ADME Properties Prediction

A staggering 40% of promising drug candidates stumble in clinical trials due to unfavorable ADME properties [21]. In silico ADME prediction can quickly evaluate the drug-likeness of a compound by calculating its pharmacokinetic parameters and physicochemical properties. This powerful tool can considerably reduce the amount of consumed time and resources during the overall drug development process. We utilized the QikProp module of Schrödinger to assess the ADME profiles of hit compounds.

### 3.4. Molecular Dynamic (MD) Simulations

To study the binding mode and stability of KRAS^G12D^ with the most potent lead compounds, we employed MD simulations. The initial KRAS^G12D^-MRTX1133 complex structure was sourced from the Protein Data Bank. The structures of KRAS^G12D^ complexed with the most potent compounds were obtained from Glide docking. For these, we performed 200 ns MD simulations using AMBER 22 to assess their stability. The restrained electrostatic potential (RESP) calculated by the Hartree–Fock (HF) method with a 6–31G* basis set in the Gaussian 09 package was used to fit the partial charges for the compound. The ff14SB force field and the general AMBER force field (gaff) were used for KRAS^G12D^ and the compound, respectively. The complex was solvated in TIP3PBOX at a distance of 10 Å to the boundary. After adding ions to neutralize the systems, the systems were minimized, heated, and equilibrated. Multiple MD procedures were employed to equilibrate the system. Finally, 200 ns MD simulations at 300 K with 1.0 atm in an NPT ensemble were performed. The trajectory analysis was performed via the *cpptraj* (version 6.4.4) module in AMBER 22. The binding free energy of each compound was determined by the molecular mechanics generalized Born surface area (MMGBSA) method [22,23].

## 4. Conclusions

In conclusion, we integrated a computational approach, combining structure-based virtual screening and molecular dynamics simulations, to identify and characterize potential noncovalent inhibitors targeting the challenging KRAS^G12D^ mutation. Over 1.7 million compounds were screened resulting in 15 promising molecules for MD analysis. MD simulations provided valuable insights into the stability, binding kinetics, and specificity of the identified inhibitors. Among the compounds, compound **1307165** emerged as a particularly noteworthy candidate, demonstrating a substantial ΔG and stable interactions with the KRAS^G12D^ protein. The interaction maps presented in Figure 6 underscored the importance of hydrogen bonds in stabilizing ligand-protein complexes. Compound **1307165** exhibited rich hydrogen bonds and a salt bridge, emphasizing its potential as a robust inhibitor. Based on the structure-based interaction analysis, this work demonstrated that a good KRAS^G12D^ inhibitor should include some amine groups to enable the possibility of forming salt bridges and hydrogen bonds. This would be a good strategy to drug the undruggable KRAS^G12D^. While the study provides promising candidates for further exploration, it is crucial to acknowledge that all these candidates are not optimized. Future research endeavors could focus on refining these structures and computational models. These findings offer hope for the development of targeted therapies to address the unmet need for cancer caused by the KRAS^G12D^ mutation. This research sets the stage for continued exploration and innovation in the field.

## Figures and Tables

**Table 1:** The docking scores of hit compounds were obtained from the Glide XP docking protocol.

Compound	Docking Scores (kcal/mol)	Compound	Docking Scores (kcal/mol)
MRTX1133	−12.18	**274371**	−8.72
**485643**	−10.11	**1121521**	−8.49
**1002187**	−9.71	**347398**	−8.48
**1166303**	−9.31	**1125857**	−8.48
**1243333**	−9.19	**1251964**	−8.45
**502065**	−9.05	**1509470**	−8.35
**294749**	−9.04	**1185436**	−8.21
**1079487**	−9.02	**909401**	−8.11
**1307165**	−9.00	**1205378**	−8.11
**1280969**	−8.96	**1292268**	−8.04
**1194622**	−8.87		

**Table 2:** Pharmacokinetic profile of the hit compounds.

Compound	Molecular Weight	logP (o/w) ^a^	logS ^b^	Caco ^c^	PMDCK ^d^	Percent Human Oral Absorption ^e^
MRTX1133	600.64	5.59	−6.77	51.67	84.37	64.45
**485643**	495.68	3.10	−2.26	110.38	119.74	81.66
**1002187**	485.55	4.74	−4.69	839.28	2372.93	100.00
**1166303**	423.56	2.77	−3.05	243.47	157.30	85.87
**1243333**	341.39	2.26	−4.37	318.84	238.58	84.97
**502065**	445.56	3.79	−4.57	187.40	145.95	89.84
**294749**	386.49	1.61	−2.38	53.46	69.44	67.30
**1079487**	362.47	3.43	−3.41	151.03	139.69	86.00
**1307165**	448.61	3.56	−3.40	557.12	501.26	96.95
**1280969**	463.97	4.36	−6.60	715.90	1315.61	100.00
**1194622**	450.63	3.33	−4.48	60.07	47.50	78.29
**274371**	415.42	2.57	−4.48	25.02	15.63	67.02
**1121521**	327.39	1.57	−3.15	103.17	46.99	72.18
**347398**	466.04	3.48	−2.98	9.42	676.19	64.75
**1125857**	384.50	2.81	−4.32	722.39	1305.50	94.58
**1251964**	471.01	4.47	−4.53	15.08	629.79	74.21
**1509470**	395.93	4.06	−4.24	119.25	149.77	87.89
**1185436**	404.47	2.42	−4.05	442.24	348.33	88.44
**909401**	427.50	3.61	−5.02	167.70	79.44	87.88
**1205378**	379.50	3.35	−4.60	392.49	199.16	92.96
**1292268**	443.55	4.06	−5.38	329.09	164.63	95.78

^a^ Predicted octanol/water partition coefficient log P (acceptable range −2.0 to 6.5). ^b^ Predicted aqueous solubility log S in mol/L (acceptable range: −6.8 to 0.5). ^c^ Predicted Caco-2 cell permeability in nm/s (acceptable range: > 50). ^d^ Predicted apparent MDCK cell permeability in nm/s (acceptable range: > 40). ^e^ Percentage of human oral absorption (acceptable range: > 60%).

**Table 3:** Ligands and their average binding free energy with KRAS^G12D^.

Compound	MMGBSA ΔG (kcal/mol)	Compound	MMGBSA ΔG (kcal/mol)
MRTX1133	−60.58	**1292268**	−20.39
**1307165**	−41.94	**1185436**	−18.34
**1194622**	−31.71	**1002187**	−17.84
**1166303**	−30.47	**1121521**	−16.01
**502065**	−27.81	**1509470**	−15.02
**1243333**	−26.11	**1280969**	−13.39
**909401**	−25.33	**1079487**	−10.02
**294749**	−24.63	**1125857**	−5.42
**485643**	−22.59	**1205378**	−1.68

## Data Availability

All data generated or analyzed during this study are included in the article.

## Supplementary Materials

The following are available online at https://www.mdpi.com/article/10.3390/molecules29061229/s1, Figure S1: The initial (white CPK representation) and the final (at 200 ns, shown in green CPK representation) pose at the docking pocket of 909401(a), 294749(b), 485643(c), 1292268(d), 1185436(e) and 1002187(f). The protein shows in ribbon colored by residue position; Figure S2: The initial (white CPK representation) and the final (at 200 ns, shown in green CPK representation) pose at the docking pocket of 1121521(a), 1509470(b), 1280969(c), 1079487(d), 1125857(e) and 1205378(f). The protein shows in ribbon colored by residue position; Figure S3: The RMSD of protein, ligand(MOL), complex and ligand aligned to the docking packet (MOL-PKT) of MRTX1133(a), 1307165(b), 1194622(c), 1166303(d), 502065(e) and 1243333(f). The configuration is save every 0.1 ns during 200 ns MD simulations. 2000 frames were save for every compound; Figure S4: The RMSD of protein, ligand(MOL), complex and ligand aligned to the docking packet (MOL-PKT) of 909401(a), 294749(b), 485643(c), 1292268(d), 1185436(e) and 1002187(f). The configuration is save every 0.1 ns during 200 ns MD simulations. 2000 frames were save for every compound; Figure S5: The RMSD of protein, ligand(MOL), complex and ligand aligned to the docking packet (MOL-PKT) of 1121521(a), 1509470(b), 1280969(c), 1079487(d), 1125857(e) and 1205378(f). The configuration is save every 0.1 ns during 200 ns MD simulations. 2000 frames were save for every compound.
