# Supplementary material for: Discovery of Novel Noncovalent KRAS G12D Inhibitors through Structure-Based Virtual Screening and Molecular Dynamics Simulations
Source: Molecules. 2024 Mar 10;29(6):1229. doi: 10.3390/molecules29061229 (PMC10975070; doi:10.3390/molecules29061229)
Supplement: Supplementary file 1 [file molecules-29-01229-s001.zip › molecules-2825723-supplementary.pdf]

Supplementary materials for Discovery of Novel Noncovalent KRAS G12D Inhibitors through Structure-Based Virtual Screening and Molecular Dynamics Simulations

Zhenya Du [1,2], Gao Tu [1], Yaguo Gong [1], Xiangzheng Fu [1], Qibiao Wu [1,*] and Guankui Long [3,*]

1 State Key Laboratory of Quality Research in Chinese Medicine, Dr. Neher's Biophysics Laboratory for Innovative Drug Discovery, Macau Institute for Applied Research in Medicine and Health, Faculty of Chinese Medicine, Macau University of Science and Technology, Macao 999078, China; 2009853qct30001@student.must.edu.mo (G.T.); gongyglab@gmail.com (Y.G.); fxz326@hnu.edu.cn (X.F.)

2 Teaching and Research Department of Public Medical Courses, School of Nursing, Guangzhou Xinhua University, Guangzhou 510520, China

3 School of Materials Science and Engineering, National Institute for Advanced Materials, Renewable Energy Conversion and Storage Center (RECAST), Nankai University, Tianjin 300350, China

* Correspondence: qbwu@must.edu.mo (Q.W.); longgk09@nankai.edu.cn (G.L.)

Citation: Du, Z.; Tu, G.; Gong, Y.; Fu, X.; Wu, Q.; Long, G. Discovery of Novel Noncovalent KRAS G12D Inhibitors through Structure-Based Virtual Screening and Molecular Dynamics Simulations. *Molecules* **2024**, *29*, 1229. https://doi.org/10.3390/molecules29061229

Academic Editor: Rachid Skouta

Received: 30 December 2023
Revised: 18 February 2024
Accepted: 8 March 2024
Published: 10 March 2024

Copyright: © 2024 by the authors. Licensee MDPI, Basel, Switzerland. This article is an open access article distributed under the terms and conditions of the Creative Commons Attribution (CC BY) license (https://creativecommons.org/licenses/by/4.0/).

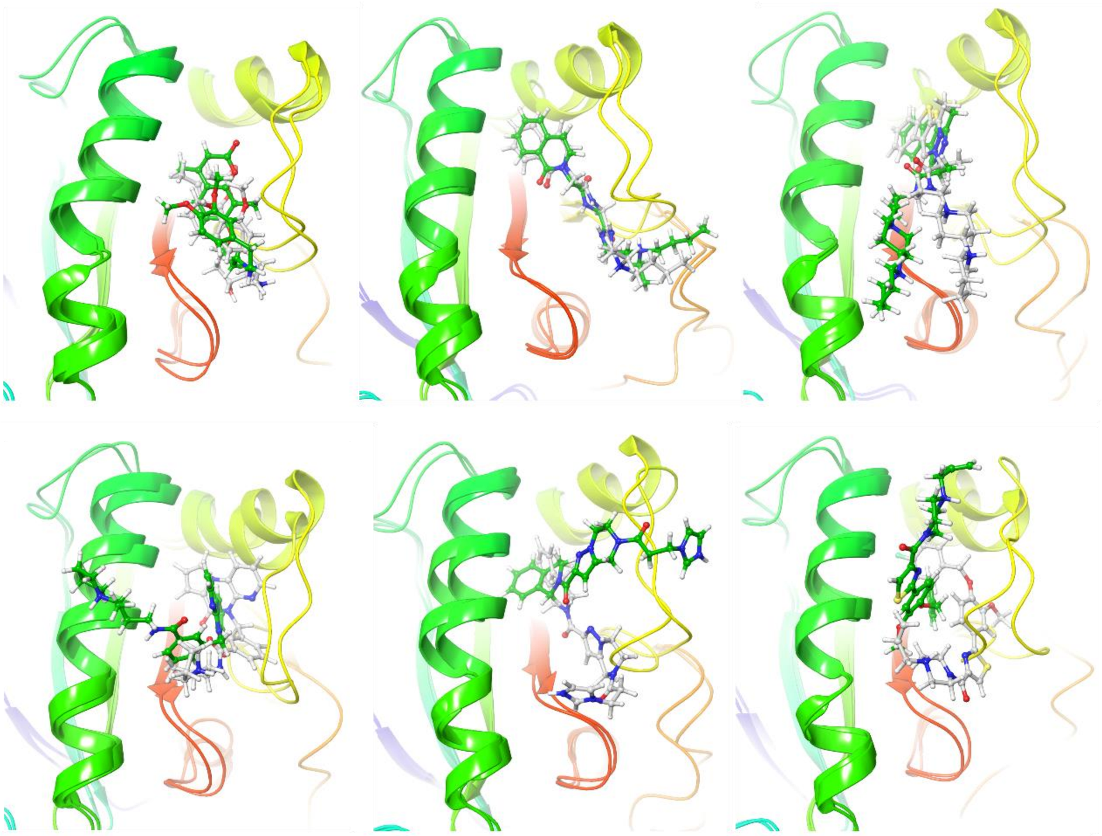

Figure S1. The initial (white CPK representation) and the final (at 200 ns, shown in green CPK representation) pose at the docking pocket of 909401(a), 294749(b), 485643(c), 1292268(d), 1185436(e) and 1002187(f). The protein shows in ribbon colored by residue position.

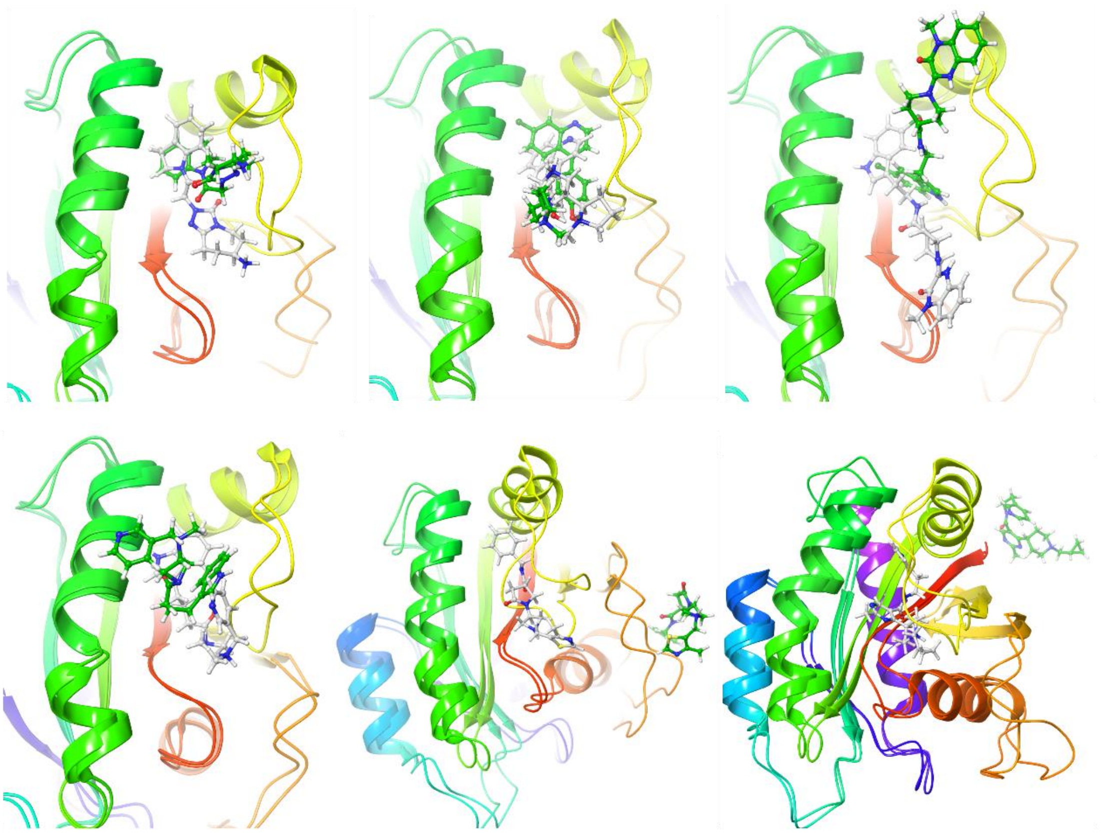

Figure S2. The initial (white CPK representation) and the final (at 200 ns, shown in green CPK representation) pose at the docking pocket of 1121521(a), 1509470(b), 1280969(c), 1079487(d), 1125857(e) and 1205378(f). The protein shows in ribbon colored by residue position.

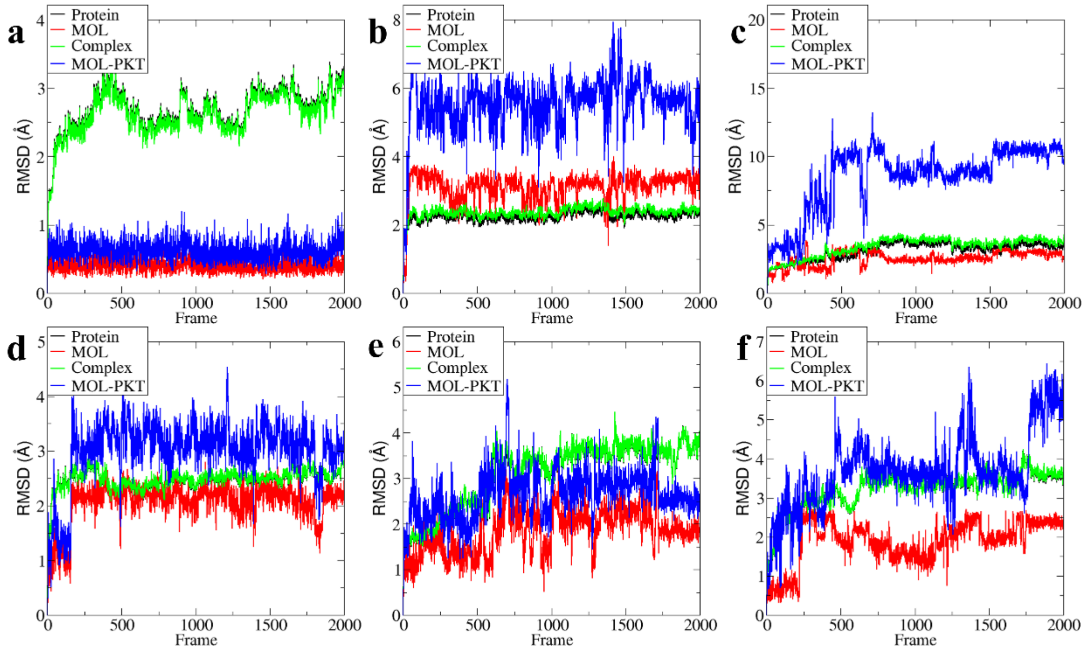

Figure S3. The RMSD of protein, ligand(MOL), complex and ligand aligned to the docking packet (MOL-PKT) of MRTX1133(a), 1307165(b), 1194622(c), 1166303(d), 502065(e) and

1243333(f). The configuration is save every 0.1 ns during 200 ns MD simulations. 2000 frames were save for every compound.

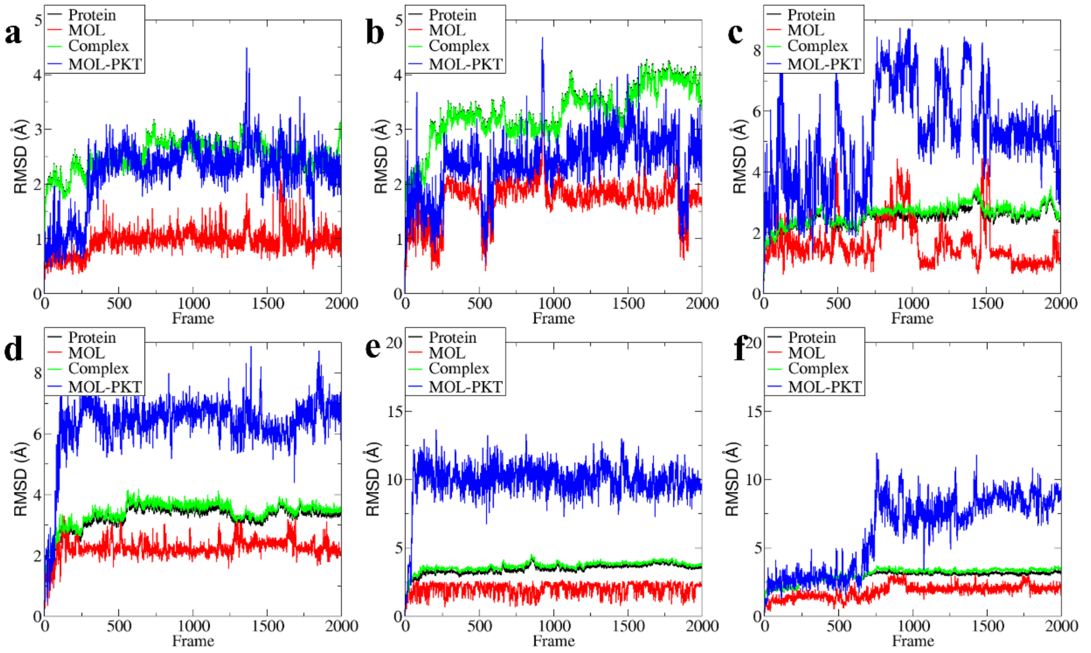

Figure S4. The RMSD of protein, ligand(MOL), complex and ligand aligned to the docking packet (MOL-PKT) of 909401(a), 294749(b), 485643(c), 1292268(d), 1185436(e) and 1002187(f). The configuration is save every 0.1 ns during 200 ns MD simulations. 2000 frames were save for every compound.

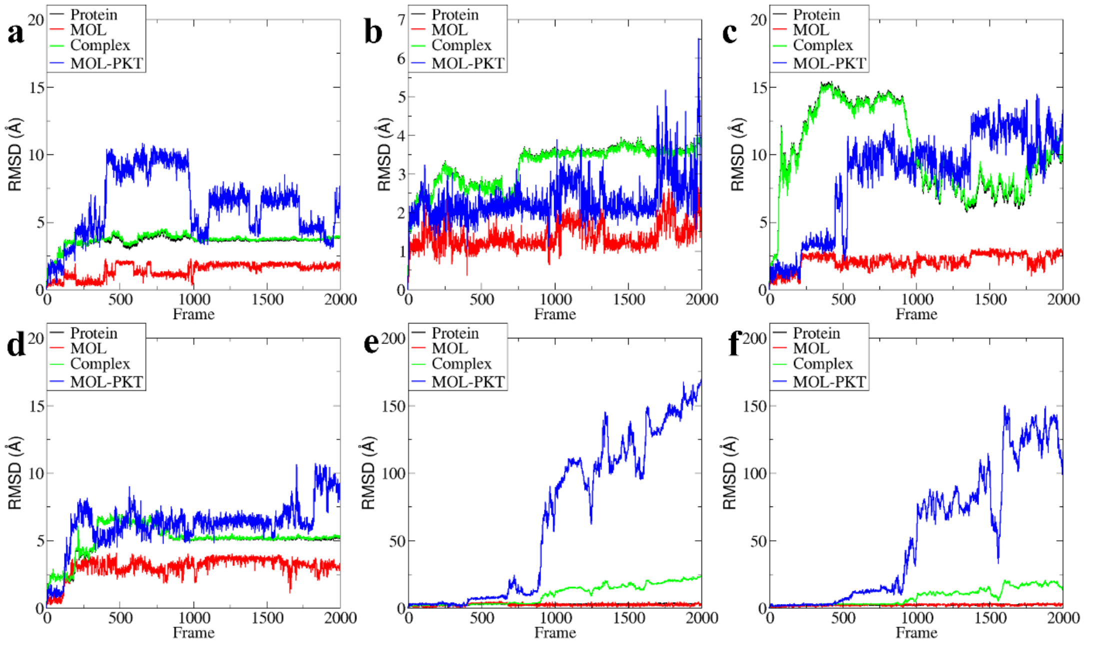

Figure S5. The RMSD of protein, ligand(MOL), complex and ligand aligned to the docking packet (MOL-PKT) of 1121521(a), 1509470(b), 1280969(c), 1079487(d), 1125857(e) and 1205378(f). The configuration is save every 0.1 ns during 200 ns MD simulations. 2000 frames were save for every compound.
